# Diversity, trait preferences, management and utilization of yams landraces (*Dioscorea* species): an orphan crop in DR Congo

**DOI:** 10.1038/s41598-022-06265-w

**Published:** 2022-02-10

**Authors:** Idris I. Adejumobi, Paterne A. Agre, Didy O. Onautshu, Joseph G. Adheka, Mokonzi G. Bambanota, Jean-Claude L. Monzenga, Joseph L. Komoy, Inacio M. Cipriano

**Affiliations:** 1 Department of Biotechnology, Faculty of Science, University of Kisangani, Kisangani, Democratic Republic of the Congo; 2 International Institute of Tropical Agriculture, Ibadan, Nigeria; 3 Institut Facultaire Des Sciences Agronomiques de Yangambi (IFA-YBI) à BP 1232, Kisangani, Democratic Republic of the Congo

**Keywords:** Genetics, Plant sciences

## Abstract

Yam (*Dioscorea* spp.) is cultivated in many villages of DR Congo as a means to sustain food security and alleviate poverty. However, the extent of the existing diversity has not been studied in details thus, considered as an orphan. A survey covering 540 farmers in 54 villages was conducted in six major yam growing territories covering three provinces in DR Congo to investigate the diversity, management and utilization of yam landraces using pre-elaborate questionnaires. Subject to synonymy, a total of 67 landraces from five different species were recorded. Farmers’ challenges limiting yam production were poor tuber qualities (69%), harvest pest attack (7%), difficulty in harvesting (6%), poor soil status (6%). The overall diversity was moderate among the recorded yam germplasm maintained at the household level (1.32) and variability exist in diversity amongst the territories and provinces. Farmers’ in territories of Tshopo and Mongala provinces maintained higher level of germplasm diversity (2.79 and 2.77) compared to the farmers in territories of Bas-Uélé (1.67). Some yam landraces had limited abundance and distribution due to loss of production interest in many villages attributable to poisons contained hence, resulting in possible extinction. Farmers’ most preferred seed source for cultivation were backyard (43%) and exchange with neighboring farmers (31%) with the objective of meeting food security and generating income. In villages where yam production is expanding, farmers are relying on landraces with good tuber qualities and high yield even though they are late maturing. This study revealed the knowledge of yam landrace diversity, constraints to production and farmers’ preferences criteria as a guide for collection and conservation of yam germplasm for yam improvement intervention.

## Introduction

Yam is a crop of major economic and cultural importance in sub-Saharan Africa where about 95% of the global production resides^1–4^. The yam belt of West and Central Africa is identified to be the principal areas of production^2,5,6^. The importance of yam has been reported in ensuring food security and enhancing livelihood systems of millions of people in sub-Saharan Africa^7^. For decades, yam as food source and cash crop has been understudied and underutilized and often referred to as orphan crop by researchers^8,9^. However, following the establishment of research institutions such as the International Institute of Tropical Agriculture (IITA), yam has gained substantial research attention in recent decades^10^. Thus, substantial progress has been made in understanding the origin, domestication, phylogeny, diversity and production of yam^10–13^.

The substantial research attention gained by yam has however to a large extent not included the central African yam germplasm as it is for west Africa. Very little is known about the status of yams in DR Congo compared to other sub-Saharan African countries. This has led to the perception that yam is not an important food crop in DR Congo as compared to other staple starch foods (maize, cassava, and sweet potato). These staple starch foods have gained substantial research attention in understanding the varietal diversity and selection: cereals^14–16^; cassava^17,18^ in DR Congo. Consequently increasing their adoption and productivity while yams that is native to the country^19^ remains neglected.

Yams are hardly known to the scientific community in DR Congo and in most cases referred to as orphan crop as no one cares for the existence. There has been no systematic study on diversity, production and use of yams in DR Congo. Although brief and passing remarks are available in the more general references^20–22^. Magwe´-Tindo et al.^20^ reported DR Congo as one of the countries with high diversity of wild yam alongside some other Central African and West African countries. Siqueira et al.^21^ reported the DNA fingerprinting performance of a *Dioscorea alata* landrace locally called “Bira” in Brazil. This landrace was introduced into the Campinas Agronomic Institute" (IAC) yam germplasm in Sao-Paulo (Brazil) from DR Congo in 1949^21^ alongside other yam accessions in the region. Bukatuka et al. reported the performance of five *Dioscorea* species (*D. alata, D. bulbifera, D. dumetorum, D. burkilliana*, and* D. praehensilis*) with respect to bioactivity and nutritional values. These authors concluded that the studied species showed good antioxidant and anti-hyperglycemic properties as well as high nutritive value. Thus, could be promoted as functional foods in DR Congo. These reports indicate that yam is widely cultivated in DR Congo, and is amongst the main root and tuber crops grown by subsistence farmers in the forest zone regions of the country.

However, the extent and distribution of the available inter and intraspecific diversity is poorly investigated. In situations where documented data are hardly available as the case in DR Congo, the local farmer is the first source of information to initiate diversity studies. Farmers’ perception of local varieties is of utmost importance because it is not only the unit of diversity they recognize but also the unit they actually manage and conserve^23^. Following previous findings^20^, it is worth understanding the landarce diversity of yam species (cultivated and wild relatives) in DR Congo to guide possible future collection and conservation of yam germplasm as well as provide useful information for future yam improvement program in the country. This study forms part of a larger objective to characterize yam genetic diversity in DR Congo. It aims to investigate the diversity of yam landraces and to describe how the landrace varieties are selected, managed and utilized by local farmers.

## Materials and methods

### Description of study area

**Figure 1:**
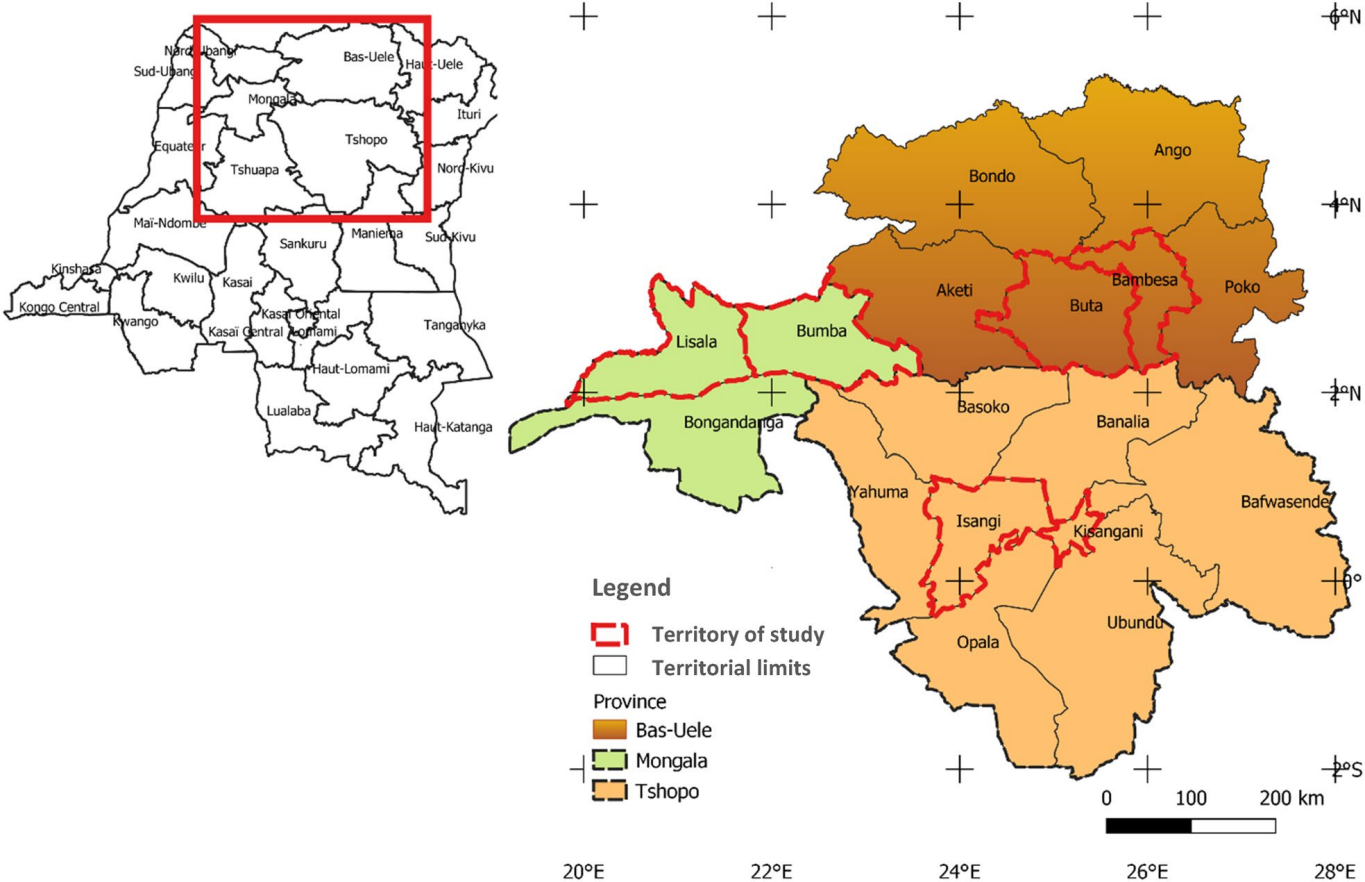
Map of DR Congo showing the six territories from the three provinces representing the study area. The map was constructed using open access QGIS software version 3.16. (https://www.qgis.org/en/site/about/index.html).

Eco-geographical and cultural similarities and production capacity were the major consideration for selection of the study areas. Following these consideration, three Administration provinces (modern map) constituting six territories: Bas-Uélé (Bambesa and Buta territories), Mongala (Bumba and Lisala territories), and Tshopo (Kisangani and Isangi territories) were selected for the study (Fig. 1) the map was constructed using the open source QGIS software version 3.16. (https://www.qgis.org/en/site/about/index.html) accessible on December 2021. Bas-Uélé province is characterized by the forest and savannah vegetation while Tshopo and Mongala provinces are characterized by the forest vegetation. Rainfall pattern is all year round and the forest vegetation forms the largest part of the vegetation in DR Congo where majority of the farming activities occur. The major food crops cultivated across the study areas include cereals (maize, rice etc.), root and tubers (cassava, yam, potatoes etc.), legumes (cowpea, pigeon pea etc.), oilseeds (peanut and soya) and fruits (banana and plantain) (Sup Table 1). Yam is locally called “Mboma” in Lingala language and “Biama” in Swahili language. These two languages form the major dialects in the study areas.

### Sampling technique and data collection

Prior to the survey, the Inspection Provinciale de l’Agriculture, Kisangani, Tshopo province was visited. Discussion was held with the Province Agricultural Inspector (Augustine Emmanuel Milabo Likele) to identify the major yam production areas in DR Congo. A total of 14 provinces with production capacity greater than 10,000 tons were identified using the national statistical data on yam^24^ from where the study areas was selected. In each province, two territories were selected in similar manner giving a total of six territories. In each territory, nine villages were selected based on yam production consistency and giving a total of fifty-four villages used for this study. Per village, a total of 10 farm families were surveyed and their farms were visited for observation on the type of yam species being cultivated following the willingness of the farmers. A total of 540 farmers who cultivate yam either as sole-cropping or intercropped with other crops were individually interviewed using a pre-elaborated questionnaire with the aid of local translators. Prior to commencement of the survey, Scientific research attestation was requested and approved by the head of the scientific research committee, faculty of sciences, University of Kisangani. The attestation was an approval from the University requesting the full support of the civil administrative authorities including the military in providing assistance in any way necessary to the success of the survey in the study areas.

During the survey, data were collected on socio-demographic characteristics, yam landrace diversity and management, crop husbandry and seedyam production system, and yam utilization. Socio-demographic characteristics include information on age, gender, educational status, family size, farming experience in years, primary occupation, and farm size. Yam landrace diversity and management includes information on number of landrace varieties, farmer preference criteria, and the diversity management. Crop husbandry and seed yam production system include information on cultivation practices, production and conservation constraints, harvesting, and seed tuber farming. After the interview, the cultivated yam and the wild yam related species were collected in with the permission of the farmers’ as stated in letter issues by the University of Kisangani. This collection is meant for landrace characterization to be done in later studies. In addition to this, all the experiments carried out in this study were performed in accordance with relevant guidelines and regulations in DR Congo. During the survey, informed consent was obtained from all subjects and legal guardian(s) for participants less than 18 years old involved in this study.

### Statistical analysis

Descriptive statistics was used in generating summary tables and means were tested for significance using 95% confidence interval where necessary. Spearman’s correlation was used to assess the relationship among the socio-demographic characteristics and the yam diversity maintained at the household level using corrplot library R package^25^. Shannon-Weiner diversity index (H’)^26^, species richness (number of unique yam landrace in an area) and equitability (E) were used to quantify the diversity of yam at the village, territory and province levels as shown below:

$${\text{H}}^{\prime } = \sum\limits_{i = 1}^{N} {pi \times ln\left( {pi} \right)}$$

where *N* is the number of yam species in villages, territories and provinces, pi the proportional abundance of the ith yam species.

$$E = \frac{{H^{\prime}}}{{H_{\max } }},\quad where\;H_{\max } = ln \left( {landrace\;richness} \right).$$

where *H*′ is Shannon diversity index and *H*_max_ represent the theorectical maximum diversity.

Subject to synonymy/homonymy, a data base was constructed by considering the unique landrace morphotypes at the village level based on the farmers’ identified agronomic and tuber quality traits and yam species. These traits include; tuber flesh oxidation status after cooking rated 1–3 (1 = no change in colour; 2 = slightly darken after cooking; 3 = heavily darken after cooking), tuber taste rated 1–3 (1 = sweet; 2 = intermediate; 3 = bitter), tuber flesh colour rated 1–9 (1 = white; 2 = cream-white; 3 = yellow; 4 = purple; 5 = purplish-white; 6 = creamy; 7 = brownish-white; 8 = deep purple; 9 = orange), tuber shape rated 1–5 (1 = oval; 2 = spherical; 3 = cylindrical; 4 = oblong; 5 = irregular), maturity duration rated 1–3 (1 = early “less than 8 months”; 2 = intermediate “8–10 months”; 3 = late “greater than 10 months”) and yam species rated 1–5 (1 = *D. rotundata*; 2 = *D. cayenensis*; 3 = *D. alata*; 4 = *D. dumetorum*; 5 = *D. bulbifera*). This data base was used to define a genetic distance matrix were the data was log transformed and scaled. Generated matrix was then subjected to clusted analysis based Ward method using UPGMA and the relatedness among the unique landrace was then visualized using cluster package in R^27^ was estimated.

## Results

### Sociodemographic characteristics of the study areas

Among the 540 farmers, 80.60% were male while 19.40% were female with an average experience in yam production being 13 years. Besides, 1.30% of the farmers surveyed were teenagers (< 20 years), 73.30% were adults class (20–50 years) and 25.40% were old people (> 50 years). Secondary and primary educations (59.80% and 34.60%, respectively) were the most common form of education. The major activity of the farmers is farming (98.70%). The average family size of the survey participants was approximately seven members with a minimum of one member and a maximum of 24 members in the case of extended family. The average farm size was 1.49 hectare with a recorded minimum farm size of 0.10 hectare and maximum farm size of 15 hectares. The average size of yam field under cultivation was 0.10 hectare with a maximum of 1.50 hectares. With respect to yam cultivation relative to other crops (food and cash), yam took an approximate of 7% of the total available land for crop cultivation in general (Table 1).

**Table 1:** Sociodemographic analysis of the survey territories.

Variables	Modalities	Territory						Total study area (n = 540)
		Bambesa (n = 90)	Bumba (n = 90)	Buta (n = 90)	Isangi (n = 90)	Kisangani (n = 90)	Lisala (n = 90)	
Gender (%)	Male	83.30	93.30	94.40	66.70	76.70	96.70	80.60
	Female	16.70	6.70	5.60	33.30	23.30	3.30	19.40
Education level (%)	No formal education	3.30	6.70	2.20	2.20	8.90	2.20	4.30
	Primary	44.40	34.40	50.00	27.80	24.40	26.70	34.60
	Secondary	52.20	57.80	46.60	70.00	63.30	68.90	59.80
	Tertiary	–	1.10	1.10	–	3.30	2.20	1.30
Major occupation (%)	Farming	97.80	96.70	100.00	98.90	100.00	98.90	98.70
	Non-farming	2.20	3.30	–	1.10	–	1.10	1.30
Age range (year)	Less than 20	2.20	1.10	–	1.10	–	3.30	1.30
	20–30	27.80	17.80	26.70	31.10	22.20	22.20	24.60
	31–40	26.70	33.30	31.10	21.10	32.20	27.80	28.70
	41–50	18.90	27.80	18.90	15.60	11.10	27.80	20.00
	Greater than 50	24.40	20.00	23.30	31.50	34.40	18.90	25.40
Family size	Average	6.86	8.14	7.40	7.21	7.34	8.47	7.60
	Minimum	1.00	1.00	1.00	1.00	2.00	3.00	1.00
	Maximum	22.00	21.00	19.00	24.00	18.00	19.00	24.00
Respondent position	Family head	82.20	94.40	73.30	73.30	76.70	96.70	82.80
	Family member	17.80	5.60	26.70	26.70	23.30	3.30	17.20
	Distant member	–	–	–	–	–	–	–
Yam field size (ha)	Average	0.01	0.41	0.03	0.05	0.07	0.03	0.10
	Minimum	0.002	0.000	0.001	0.010	0.010	0.001	0.000
	Maximum	0.20	1.50	1.00	0.50	1.00	0.50	1.50
Farm size (ha)	Average	0.98	2.65	1.33	1.31	1.27	1.40	1.49
	Minimum	0.10	0.50	0.50	0.40	0.25	0.50	0.10
	Maximum	2.50	11.00	4.00	10.00	12.00	15.00	15.00
Land use (%)	Yam	1.44	15.86	1.98	3.65	5.35	2.03	6.62
	Other crops	98.56	84.14	98.02	96.35	94.65	97.97	93.38
Yam experience (year)	Average	10.81	8.57	11.48	15.84	14.09	13.89	12.46
	Minimum	1.00	1.00	1.00	1.00	1.00	1.00	1.00
	Maximum	50.00	35.00	45.00	63.00	43.00	47.00	63.00

### Constraints to yam production in DR Congo

Generally, tuber quality forms the highest proportion (69%) of the farmers’ constraints to yam production. The traits reported by farmers under the tuber quality were poor postharvest shelf life (30.58%), high tuber flesh oxidation (14.54%), poor taste (~ 14%) and rapid hardiness of tubers (10%) (Table 2).

**Table 2:** Summary of constraints limiting yam production in DR Congo.

Category	Factors	Percentage of responses						
		Bambesa n = 90	Buta n = 90	Bumba n = 90	Lisala n = 90	Kisangani n = 90	Isangi n = 90	Total n = 540
Tuber quality	Poor post-harvest shelf life	32.32	23.08	19.58	31.03	47.17	30.28	30.58
	High tuber flesh oxidation	26.26	15.38	1.67	18.23	3.14	22.54	14.54
	Poor taste	27.95	17.95	–	15.27	1.26	20.42	13.81
	Rapid tuber hardiness	7.74	5.13	–	20.69	10.06	18.31	10.32
		**94.27**	**61.54**	**21.25**	**85.22**	**61.63**	**91.55**	**69.24**
Biotic	Pests	–	–	24.58	1.48	12.58	2.11	6.79
	Theft	–	–	15.42	0.49	–	0.7	2.77
		–	–	**40**	**1.97**	**12.58**	**2.81**	**9.56**
Abiotic	Poor soil	–	17.95	13.33	4.93	0.63	–	6.14
	Lack of storage facility	5.72	–	0.83	–	–	2.82	1.56
	Poor transportation means	–	–	1.67	–	3.77	–	0.91
	Lack of finance	–	–	1.67	–	–	–	0.28
	Work accident	–	–	0.42	–	–	–	0.07
		**5.72**	**17.95**	**17.92**	**4.93**	**4.4**	**2.82**	**8.96**
Agronomic quality	Difficulty in harvesting	–	17.95	14.17	4.93	0.63	–	6.28
	Difficulty in processing	–	–	–	2.96	–	–	0.59
	Lack of seedyam	–	–	1.67	0	–	–	0.28
	Field management difficulty	–	–	0.83	0	–	–	0.14
	Low viability of seedyam	–	–	0.42	0	–	–	0.07
		–	**17.95**	**17.09**	**7.89**	**0.63**	–	**7.26**
Marketing	Poor market price	–	–	3.75	–	18.24	–	3.67
	No market demand	–	2.56	-	–	2.52	2.82	1.32
		–	**2.56**	**3.75**	–	**20.76**	**2.82**	**4.98**

Significant values are in [bold].

The biotic factors (9.56%) followed after tuber quality. This was largely influenced by the proportion of farmers who reported harvest pests’ problem (~ 7%) while theft was only ~ 3%. The abiotic factors form the third constraint reported by farmers (8.96%). This constraint was influenced by farmers who reported poor soil (6%) compared to other factors in this category.

Agronomic quality (7.26%) was also identified by farmers as a constraint to production influenced by difficulty in harvesting (6%). The last production constraint reported by farmers was marketing (~ 5%). This forms the least of the farmers’ production constraints and is influenced by poor market price for yam tubers (~ 4%). In summary, the major factors reported by farmers as constraints to yam production in DR Congo are: poor post-harvest shelf life, high tuber flesh oxidation, poor tuber taste, rapid tuber hardiness, pests, difficulty in harvesting, poor soil, and poor market price.

Different territories have different constraints affecting yam production. For example, while tuber quality traits were identified as the primary production constraints for Bambesa (94%), Buta (~ 62%), Lisala (85%), Kisangani (~ 62%) and Isangi (~ 92%), it was not the case for Bumba (21%) rather biotic factors (40%) forms the primary constraints to production in this territory. Yams in this territory were not affected by poor taste and tuber hardiness (Table 2).

### Yam landrace diversity

**Figure 2:**
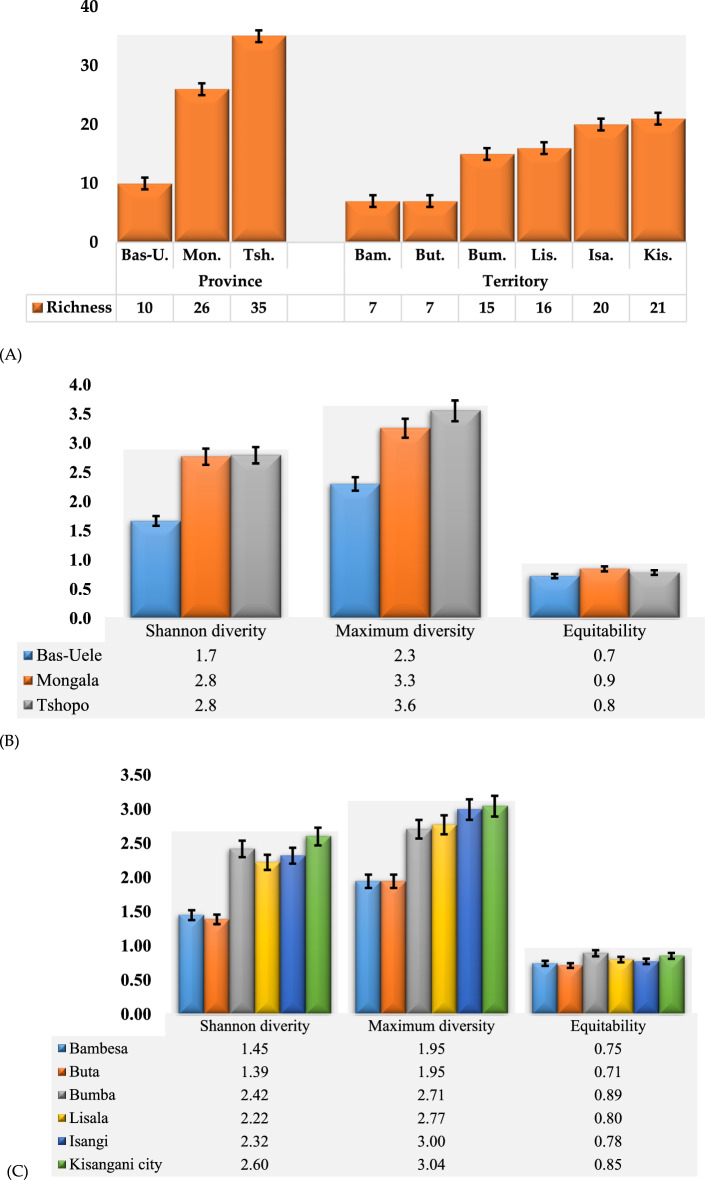
Landrace richness and diversity at the level of province and territory: landrace richness (**A**), landrace diversity at province level (**B**) and at territory level (**C**).

The mean landrace diversity expressed as Shannon index (H’) and the H_max_ (the maximum possible in study areas) were 1.32 and 1.58 respectively (Sup Table 2). Landrace richness was observed to significantly differ between the provinces. Tshopo province has the highest landrace richness (35) which is statistically different from that of Mongala (26) and Bas-Uélé (10). The territories within respectives provinces have statistically similar landrace richness however different from territories of other provinces (Fig. 2A). With respect to H’, both Tshopo and Mongala provinces had significantly higher level of landrace diversity (2.79 and 2.77 respectively) than Bas-Uélé (1.67). Similar trend was observed for H_max_ which explains the maximum diversity present in these provinces (Fig. 2B). At the level of the territory, Kisangani had the highest recorded landrace diversity (H′ = 2.60) which is significantly different from other territories but similar to Bumba. Territories of Bumba, Isangi and Lisala had similar level of landrace diversity (H′ = 2.42, 2.32 and 2.22 respectively) significantly higher than that of Bambesa and Buta (H′ = 1.45 and 1.39 respectively). With respect to H_max_, Kisangani, Isangi, Lisala and Bumba had statistically similar H_max_ (3.04, 3.00, 2.77, and 2.71 respectively) significantly higher than that of Buta and Bambesa (1.95 and 1.95 respectively) (Fig. 2C).

Considering the possibility of synonyms, the numbers of yam landraces recorded ranged from two to ten per village. The minimum number of landrace (2) was observed in the villages of Bambesa (Adiwaya, Bongenge, Dingima, and Mendigba) and Buta (Bobomale, Bonzo and Boyelia) territories while the maximum number of landrace (10) was observed in the villages of Isangi territory (Q. Bangala and Q. Lumumba) (Sup Table 2).

**Figure 3:**
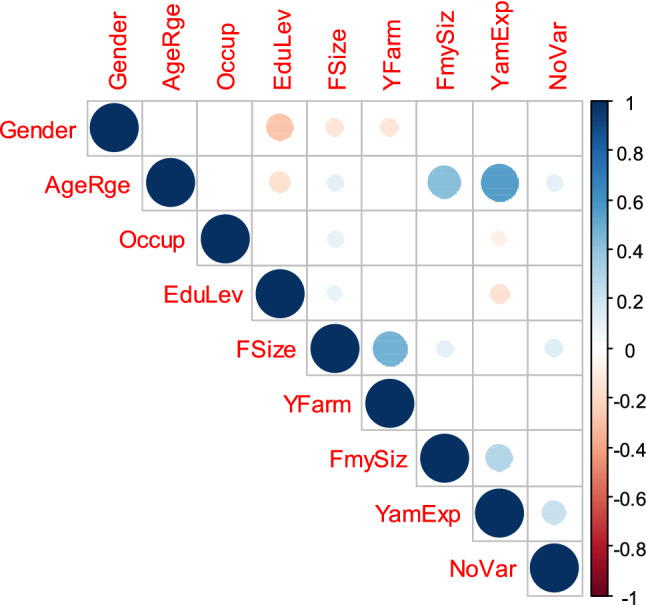
Relationship between the number of landarces at household level and socio-demographic parameters. *AgeRge* age range, *Occup* occupation, *EduLev* educational level, *FSize* farm size, *YFarm* yam farm size, *FmySiz* family size, *YamExp* yam cultivation express, *NoVar* number of landrace cultivated.

The relationship between socio-demographic characteristics and the number of landraces cultivated at individual household level is presented in Fig. 3. Yam experience in cultivation, farm size, and farmer age had significantly positive relationship with the number of landraces cultivated at household level.

**Figure 4:**
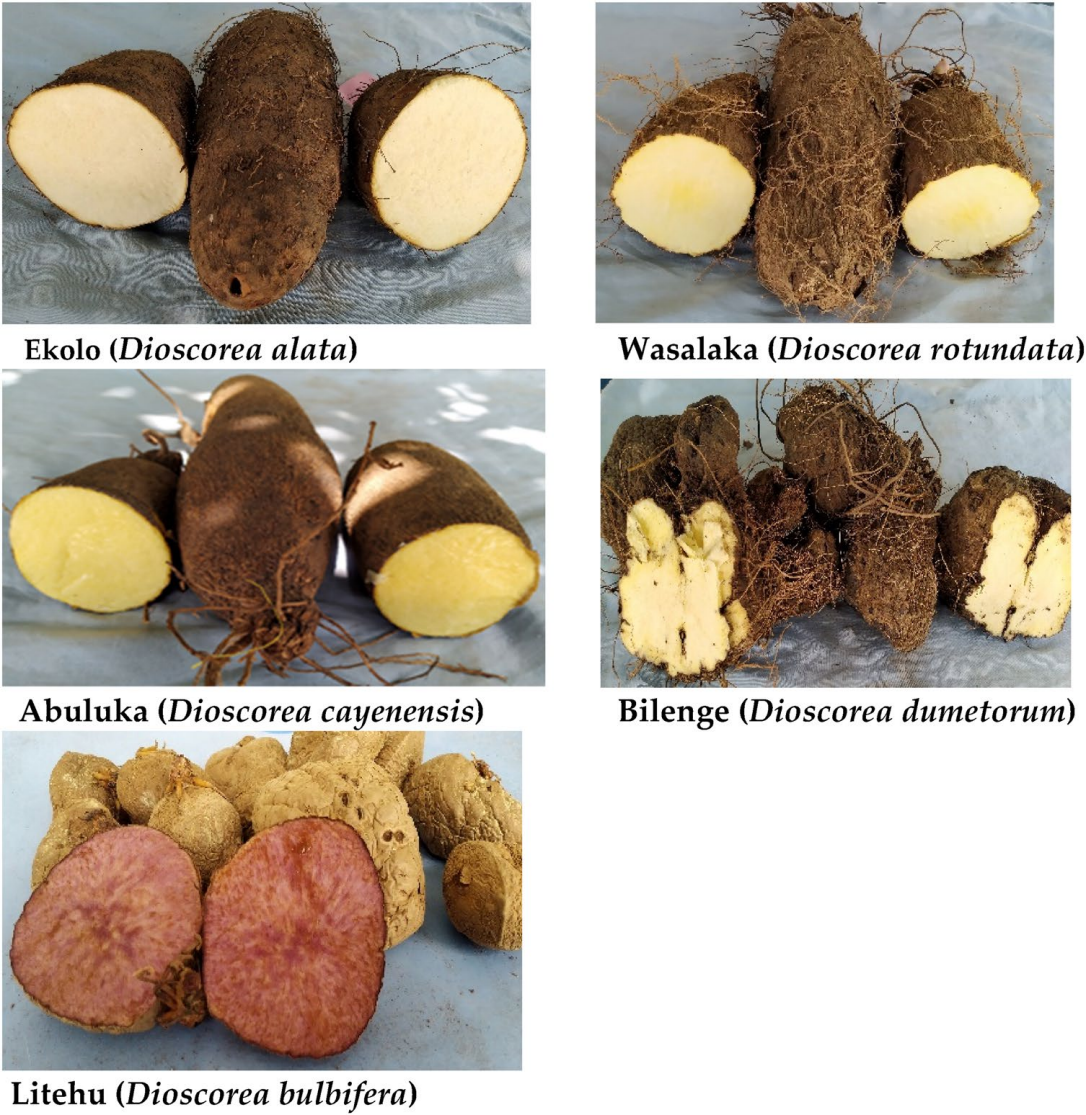
Some yam landraces from the five yam species identified across the study areas.

Throughout the entire survey period, five different species of yams (*D. rotundata, D. cayenensis, D. alata, D. dumetorum and D. bulbifera*) were recorded (Fig. 4). These species form the major landraces that were cultivated by farmers as well as in the forest in the survey areas. The quantity of landrace varieties per species also varies from one village to another with number varying from one to three (*Dioscorea rotundata, Dioscorea cayenensis, and Dioscorea dumetorum*), one to four (*Dioscorea alata*), and one to two (*Dioscorea bulbifera*). Only two villages: Quartier Bangala and Yalinga in Isangi territory (Tshopo province) were found to have all the yam species represented (Table 3).

**Table 3:** Yam species diversity across the study area.

Province	Territory	Village	E	*D. rot*	*D. ala*	*D. cay*	*D. bul*	*D. dum*	Total
Bas-Uélé	Bambesa	Adiwaya	0.44	1		1			2
		Bango	0.81	2		1			3
		Bombele	0.81	2		1			3
		Bongenge	0.86	1		1			2
		Bulusu	0.81	3		1			4
		Dingima	0.65	1		1			2
		Mendigba	0.95	1		1			2
		Mupembe	0.79	3		1			4
		Ngbonga	0.92	2	1	1			4
	Buta	Baebona	0.92	2	1	1		1	5
		Bobanabendea	0.82	2	1	1		1	5
		Bobomale	0.92	1		1			2
		Bonzo	0.78	1		1			2
		Boyelia	0.92	1		1			2
		Kumu	0.80	1	1	1			3
		Monjila	0.77	1	1	1			3
		Q.Bale	0.80	2		1		1	4
		Sombo	0.75	1	1	1			3
Mongala	Bumba	Bongolo-II	0.81		2	1		1	4
		Bonzo	0.93	1	2	3		1	7
		Botsholi-I	0.98		1	1		1	3
		Botsholi-II	0.55		2	1			3
		Yamaluka-II	0.83			3			3
		Yamoguo	0.98		1	3			4
		Yamolea-II	0.80		1	3			4
		Yanjumbu	0.81		3	1		1	5
		Yapembe	0.81	1		3			4
	Lisala	Bobi	0.92	1	2	2		1	6
		Bokutu	0.86	2	2	2		1	7
		Bosokuluki-I	0.91		2	1			3
		Bosokuluki-II	0.79	1	1	2			4
		Botukwa	0.78	1	2	2		1	6
		Dika	0.91	3	3	1		2	9
		Liweya	0.85	1	2	2		1	6
		Mapasa	0.92	2	2	1			5
		Ngunzibalele	0.91	2	2	1			5
Tshopo	Isangi	Lilanda	0.78		1	1		2	4
		Q. Bangala	0.96	1	4	1	2	2	10
		Q. Lumumba	0.83	1	3	3		3	10
		Yakako-I	0.96			1		2	3
		Yakpondi	0.91	1		1		2	4
		Yalibua	0.97		1	1		2	4
		Yalinga	0.87	2	2	1	1	2	8
		Yaondolo-II	0.96		1	1		2	4
		Yaselia	0.89	1		1		2	4
	Kisangani	Babugana	0.82	1	1	1		2	5
		Batikayafi	0.77	1	1	1		1	4
		Likenga	0.55	1	1	1			3
		Lugnunga	0.88	2	2	1		1	6
		Magbololo	0.71		1	1		2	4
		Maleke	0.79	1	3	2		1	7
		Ngenengene	0.87	1		2	1	1	5
		Ngenengene-II	0.95	1	1	1		1	4
		Osio	0.86	2		1		2	5

*E: equitability; D.rot: Dioscorea rotundata; D. ala: Dioscorea alata; D.c ay: Dioscorea cayennensis; D. bul; Dioscorea bulbifera; and D. dum: D. dumetorum.*

The maturity classification of the yam landraces in DR Congo was also assessed (Table 4). 74% of the farmers reported that the yams are of the late maturity class (> 10 months), 17% reported intermediate maturity class (8–10 months) and 9% reported early maturity class (< 8 months). At the territory level, majority of the farmers also agreed to the yams being the late maturity class except for farmers in the Kisangani. In this territory, 33% of the farmers reported early maturity class, 36% reported intermediate maturity class and 31% reported late maturity class.

**Table 4:** Yam maturity classification.

Category	Bas-Uélé Province		Mongala Province		Tshopo Province		Total n = 540
	Bambesa n = 90	Buta n = 90	Bumba n = 90	Lisala n = 90	Kisangani n = 90	Isangi n = 90	
**Average yam maturity level (%)**							
Early (< 8 months)	–	7.78	–	6.67	33.33	8.89	9.48
Intermediate (8–10 months)	3.33	18.89	10.23	17.78	35.56	15.56	16.89
Late (> 10 months)	96.67	73.33	89.77	75.55	31.11	75.56	73.67

**Figure 5:**
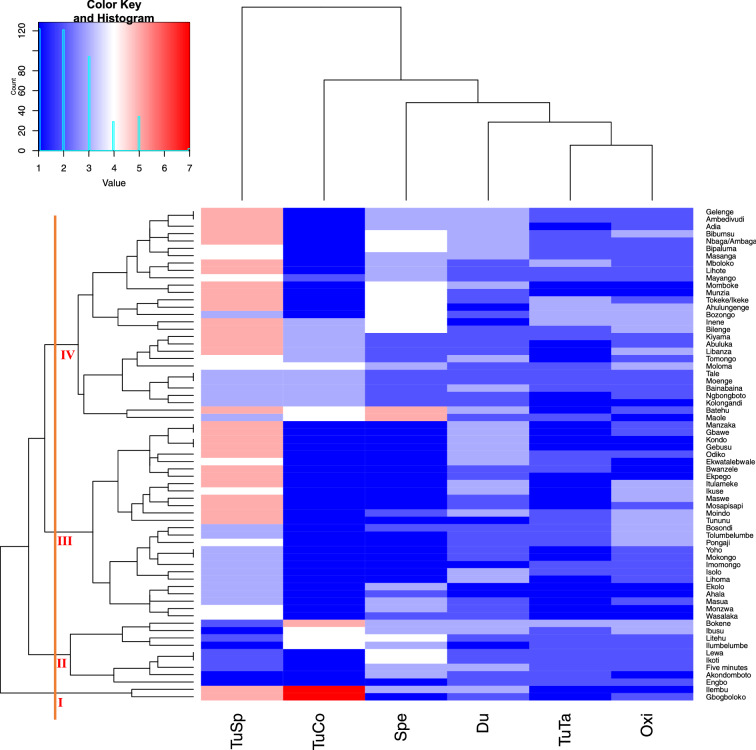
Dendrogram presenting the classification of yam (*Dioscorea* species) based tuber qualities and agronomic traits. *TuSP* tuber shape, *TuCo* tuber flesh colour, *Spe* species of yam, *Du* maturity duration, *TuTa* tuber taste after cooking, *Oxi* tuber flesh oxidation.

The relationship among the unique 67 yam landraces observed during the survey period with respect to agronomic characters (tuber shape and maturity duration), tuber quality parameters (tuber colour, tuber taste, and tuber oxidation (browning) after cooking) and yam species is represented in Fig. 5. The cluster analysis partitioned the different landrace varieties into four clusters. Cluster one consists of landraces of the *D. rotundata* and *D. alata* having irregular tuber shape, creamy flesh colour, intermediate to late maturity, sweet taste, and no-oxidation to slight browningafter cooking. Cluster two consists of landraces of the *D. rotundata*, *D. alata* and *D. dumetorum* having oval and spherical tuber shape, white and purplish-white flesh colour, early to late maturity, intermediate to bitter taste, and no-oxidation to heavy browning after cooking. Cluster three consists of landraces of the *D. rotundata*, *D. alata* and *D. cayenensis* having cylindrical and oblong tuber shape, white flesh colour, early to late maturity, sweet to intermediate taste and no-oxidation to heavy browningafter cooking. Cluster four comprised of landraces of the *D. cayenensis*, *D. alata*, *D. dumetorum* having cylindrical and oblong tuber shape, white, yellow and purple colour, early to late maturity, sweet to bitter taste, and no-oxidation to heavy browning after cooking.

Following the observed diversity of yams in the study areas, approximately 74% of the farmers also reported landraces experiencing decline in attention by farmers or extinction (Table 5). A total of 14 yam landrace names were reported by farmers that fall in this category of event. Of the proportion of farmers that reported varietal loss, 78% reported poisons as the principal reason for extinction, followed by poor tuber quality attributes (10%) and late maturity (8%).

**Table 5:** Reason leading to yam landraces loss.

Category	Bas-Uélé Province		Mongala Province		Tshopo Province		Total n = 540
	Bambesa n = 90	Buta n = 90	Bumba n = 90	Lisala n = 90	Kisangani n = 90	Isangi n = 90	
**Variety in extinction (%)**							
Positive response	100	100	71.59	100	10	60	73.6
Negative response and do not know			28.41		90	40	26.4

Reason for extinction (%)			n = 65		n = 9	n = 55	
Poisonous	100	100	23.81	95.56	55.56	94.44	78.23
Poor tuber quality	–	–	53.97	4.44	–	–	9.73
Late maturity	–	–	–	–	44.44	5.56	8.33
Lack market demand	–	–	17.46	–	–	–	2.91
Low yield	–	–	4.76	–	–	–	0.79

### Yam cultivation and cultural practices

**Figure 6:**
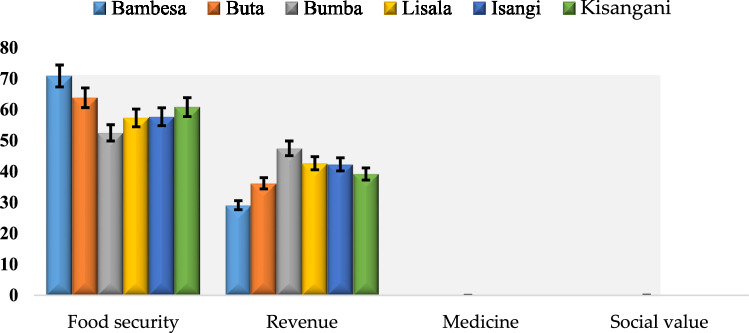
Major reasons for yam cultivation in DR Congo.

The major reason for yam cultivation presented in Fig. 6 showed that achieving food security is generally of significant priority followed by revenue in all the territories considered. With respect to food security, farmers in Bambesa showed higher preference (71%) significantly different from all other territories. Farmers in Bumba showed the least preference (52%) though statistically similar to that of Lisala and Isangi territories. With respect to revenue, farmers in Bumba showed the highest preference (47%) significantly different from other all other territories while farmers in Bambesa showed the least preference (29%) (Fig. 6).

Cropping system mostly observed in DR Congo was intercropping pattern (90%) where yam is mostly intercropped with other arable crops. Approximately 10% of the farmers practice sole cropping system (Table 6). At the province and territory level, similar trend of results was obtained except for Bumba territory where the gap between these two cropping systems was significantly reduced. In this territory, over 40% of the farmers practice sole cropping system for yams as compared to other territories where less than 15% of the farmers were observed.

**Table 6:** Cultural practices in yam farming.

Category	Bas-Uélé Province		Mongala Province		Tshopo Province		Total n = 540
	Bambesa n = 90	Buta n = 90	Bumba n = 90	Lisala n = 90	Kisangani n = 90	Isangi n = 90	
**Cropping system (%)**							
Intercropping	100	100	56.67	100	88.89	95.56	90.18
Sole cropping	–	–	43.33	–	11.11	4.44	9.82
**Staking (%)**							
Positive response	25.56	11.11	100	61.11	74.44	75.56	57.96
Negative response	74.44	88.89		38.89	25.56	24.44	42.04

	n = 23	n = 10	n = 90	n = 55	n = 67	n = 68	n = 313
**Staking period (%)**							
After 1 month	100	100	94.44	100	100	100	99.07
After 2 months	–	–	–	–	–	–	–
After 3 months	–	–	5.56	–	–	–	0.93
After 4 months	–	–	–	–	–	–	–
**Staking type (%)**							
Trellis	–	–	–	–	–	–	–
Individual	100	100	100	100	100	100	100
Group	–	–	–	–	–	–	–
**Staking material (%)**							
Wooden stick	100	100	100	100	100	100	100
Bamboo stick	–	–	–	–	–	–	–
Others	–	–	–	–	–	–	–
**Harvest signature (%)**	–	–	–	–	–	–	–
≤ 25% leaf senescence	–	–	–	–	–	–	–
50% leaf senescence	–	–	–	–	–	–	–
100% leaf senescence	100	100	100	100	100	100	100
**Number of harvest (%)**							
Once	100	100	100	100	100	100	100
Twice	–	–	–	–	–	–	–
**Activity after harvest (%)**							
Storage	100	100	75.56	88.89	85.56	82.22	88.71
Sold directly	–	–	24.44	11.11	14.44	17.78	11.30

During the production period of yam (Table 6), approximately 58% of the farmers provide staking support for their yams. At the level of the province, only 18% responded positively to supporting their yams with stakes during the production cycle in Bas-Uélé. Provinces of Mongala and Tshopo however have appreciable proportion (above 70%) of farmers that incorporated staking as part of their cultural practice. At the level of the territory, Bambesa had only ~ 26% of farmers who practiced staking, Buta had even lesser proportion (11%) of farmers. While the majority of the farmers in these territories have neglected staking, farmers in the territory Bumba have made it a necessary requirement for yam production (Table 6).

Throughout the survey, the harvest signature used by farmers is total senescence of leaves. Harvesting is only done once as farmers do not practice milking. Once harvesting of the tubers is completed, approximately 89% of the farmers prefer to go for storage of their produces while 11% of the farmers send their produce directly to the market for sale. In all the provinces and territories, at least 11% of the farmers send their produces directly from the field to the market after harvest except for the famers in the province Bas-Uélé (Bambesa and Buta territories) that prefer to go for storage (Table 6).

### Seedyam production system

**Figure 7:**
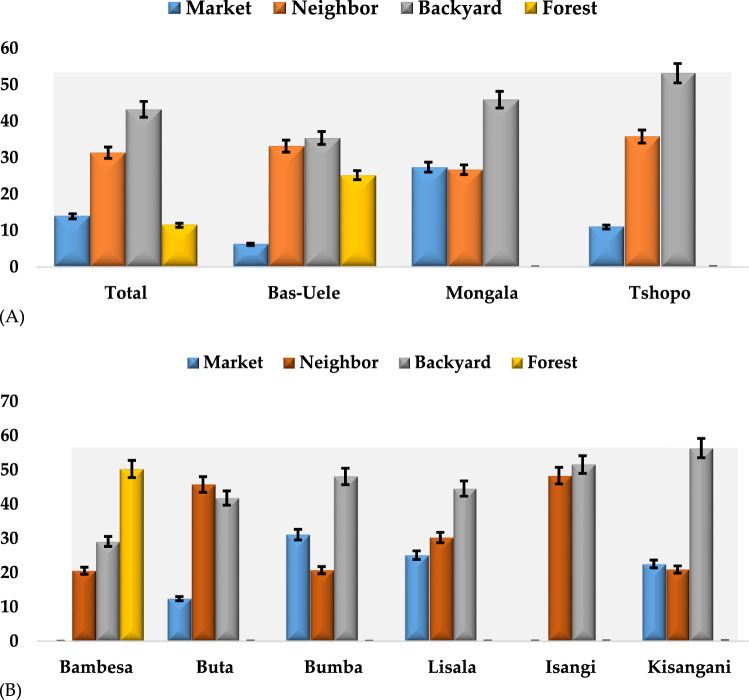
Seedyam sources in the study areas: overall and at province level (**A**), territory level (**B**).

Of four different sources of seedyam presented to the farmers, overall results showed that the backyard source (43%) (retention from previous season harvest) was the most used means to obtain seedyam. Neighbor source (31%) (exchange with neighboring farmers and friends) followed in significant ranking. Forest source (11%) is the least used means (Fig. 7A). At the level of the province, farmers in the Bas-Uélé province significantly preferred the use of Backyard source and Neighbor sources (35% and 33% respectively). Farmers in Mongala province significanty prefer the use of backyard source (46%) with neighbor and market coming as second significant preferences. Farmers in Tshopo province significantly preferred backyard source (53%) followed by neighbor source (Fig. 7A).

At the level of the territory, farmers in Bambesa statiscally preferred the use of the forest source (50%) compared to all other sources. Farmers in Buta significantly showed preference for neighbor (46%) and backyard (42%) sources. In Bumba and Lislala, significant preference was reported for backyard source (48% and 45% respectively). Farmers in Isangi territory however reported significant preference for backyard and neighbor sources (52% and 48% respectively). In Kisangani, farmers reported the significant preference for backyard sources (Fig. 7B).

### Farmers’ preference criteria for yam selection and utilization in DR Congo

The assessment of farmers’ preference criterial for yam selection and utilization in DR Congo is presented in Table 7. The preference for yams variety with good tuber quality (sweet taste and no tuber browning after cooking) was reported by 53% of the farmers, high yield preference was reported by 38% of the farmers, and preference for earliness was reported by 7% of the farmers. Thus, these three criterial accounted for over 97% of the farmers’ preference in DR Congo. At the level of the province, similar trend of results was observed across the three province however, this was not totally the case at the level of the territory. Farmers in Bambesa territory only reported the preferences for two criteria only (high yield = 50% and good tuber quality = 50%). Farmers in Buta showed preference for three criteria with importance placed on two (good tuber quality = 57% and high yield = 42%). Farmers in Bumba reported four criteria with importance placed on two criteria (high yield = 47% and good tuber quality = 47%). Farmers in Lisala reported the preference for all the traits with emphasis on good tuber quality (59%) and high yield (30%). Farmers in Kisangani reported the preference for three criteria (good tuber quality = 47%; earliness = 37%; high yield = 16%). Farmers in Isangi also reported preference for three traits with emphasis on two (good tuber quality = 56% and high yield = 42%).

**Table 7:** Farmers preference criterial and their utilization.

Category	Bas-Uélé Province		Mongala Province		Tshopo Province		Total n = 540
	Bambesa n = 90	Buta n = 90	Bumba n = 90	Lisala n = 90	Kisangani n = 90	Isangi n = 90	
**Yam selection criteria (%)**							
Good tuber quality (e.g. Taste)	50.00	57.42	47.62	59.46	46.77	55.90	52.86
High yield	50.00	41.93	47.62	30.41	16.13	41.61	37.95
Earliness	–	0.65	–	2.03	37.10	2.48	7.04
Prolong shelf life	–	–	3.87	4.05	–	–	1.32
Ease of harvesting	–	–	–	2.03	–	–	0.34
Ease of processing	–	–	1.94	2.03	–	–	0.66
**Form for consumption (%)**							
Boiled form	100.00	100.00	33.46	57.69	78.26	84.11	75.59
Pounded form	–	–	22.43	41.03	–	4.67	11.36
Grilled form	–	–	25.10	1.28	17.39	6.54	8.39
Fried form	–	–	19.01	–	4.35	4.67	4.67
Value added form (e.g. cake)	–	–	–	–	–	–	–

The major form of consumption assessed amongst the survey respondents (Table 7) showed that boiled form is the most preferred method of consumption (76%). Value addition form remain unknown to the survey respondents hence remain unexploited. With respect to the territories, farmers in Bambesa and Buta do not consume yam in any other form than boiled form which maybe attributable to the challenge of low tuber quality prevalence in these territories. Farmers in Bumba reported four forms of consumption (boiled yam = 33%; grilled yam = 25%; pounded yam = 22%; fried yam = 19). This territory has some of the best landrace varieties of yams in the survey regions. Farmers in Lisala preferred boiled form (~ 58%) and pounded form (41%). Farmers in Isangi had preference for boiled yam (84%) compared to other forms.

## Discussions

### Constraints linked to yam production in DR Congo

In general, our study reveal the presence of a moderate diversity of landrace that could support the collection and conservation of yam germplasm for future use. Though when this diversity is compared to similar findings from other yam producing countries, it is lower. The provinces (including villages) of the forest agro-ecology holds larger landrace diversity than that of the transition zone. Thus, we could infer that the variation in the number of landrcaes at household level could be attributed to agro-ecology, climatic and human factors. Increase in the diversity of yam species in the forest has also been reported by^28,29^ in Togo and^30^ in cultivated species in Ghana. Our study observed five different species of *Dioscorea*, however, we cannot totally ascertain that all the landrace morphotypes within each *D. species* are truly genetically distinct due to the possibility of linguistic polymorphism. Hence, the likelihood that this study has underestimated or overestimated the actual number of landraces cannot be ruled out. Similar studies have also reported the influence of linguistic polymorphism in bush yam in the central region of Ghana^31^; bitter yam in Benin^32,33^; and *Dioscorea* species in Southern Ethiopia^10^. The proposed challenge with linguistic polymorphism in this study could be easily clarified with further study on morphological and molecular characterization of the landraces observed.

Aside comparably lower landrace diversity to other major yam producing countries, yam production is faced with many constraints in DR Congo with the principal being tuber quality attributes of the landraces (poor post-harvest shelf life, high tuber flesh oxidation, poor taste, and rapid tuber hardiness). This has largely discouraged a lot of farmers from the cultivation of yam, affected market demand leading to poor pricing by the consumers and thus, reducing farmers profit margins. The influence of tuber quality attributes on the adoption and abandonment of yams has also been reported in many studies. For example, It was reported as a contributing factor for yam varietal loss^34^ as well as abandonment of bush yam^28^ in Togo. Other important constraints identified by this study include difficulty in harvesting, poor soil, and pest and disease.

Exisiting landrace maturity is mostly the late type. We infer this could be the consequence of the lack of genetic improvement through breeding and selection. Even with the presence of the National Agricultural Study and Research Institute (INERA) and numerous higher institutions, yams have received insignificant attention with respect to varietal improvement. Such programs could have assisted in proper collection, documentation, and conservation of yam germpalsm to prevent loss of genepools. The insignifant attention has also enhanced the loss of some landraces. Farmers reported 14 landraces that have been abandoned in cultivation and/or usage primarily due to poisons. Of the observed species *D. bulbifera* has been mostly implicated in this regard. However, not all the morphotypes of this species are poisonous as it is still being consumed by some people as observed during the survey. The question remains the proper differentiation of the morphotypes safe for consumption from the genepool. Modern breeding techniques such as detailed morphological characterization, molecular tools (DNA markers e.g. SSRs and SNPs) and DNA sequencing could help to tackle this challenge.

The practice of generating seedyam from previous harvests is a common phenomenon for seasonal yam cultivators. Farmers also engage in trade by batter (changing other food crops for seedyam for field establishment). These methods has contributed to low viability and inadequacy of seeds availability. This system is currently putting the yam producers at a disadvantage unknown to many of the farmers considering the weight of the setts for planting, lack of the knowledge and/or zeal to practice double harvesting, and high tuber losses due to poor storage. Different methods of generating seedyam have been researched and proposed to yam farmers in many yam producing countries^35,36^. Of these methods, the miniset system appeared to be most successfully adopted by farmers in many yam producing countries and thus could be attempted in DR Congo.

Farmers’ preferences for selection and utilization of yams were tuber quality attributes (good taste and non-oxidizing flesh color) and agronomic characteristics (high-yield and earliness). According to the farmers, realization of these criteria will spark a new line of interest in the mind of many farmers. Thus, establishing a yam improvement program with the objective of assisting the farmers’ should put these criteria into consideration. Similar study have also reported good tuber qualities to enhance yam marketability and cultivation^28^.

### Perspectives for yam improvement in DR Congo

Enobblement effort and seasonal cultivation by farmers have kept yam diversity in DR Congo from total loss. The establishment of yam improvement program would go a long way to providing a substancial solution to majority of the constraints linked to yam production. Such program would facilitates the collection and conservation of germpslams to prevent loss of yam gene pool, increase yam genetic diversity through hybridization and introduction, facilitates the proper characterization of yams to distinguish the consumable and the non-consumable morphotypes, and develop new and improved yams that will meet farmers’ and consumers’ requirements through selection. These have been observed in countries where yam improvement program is currently existing such as Nigeria, Ghana, etc.

Another important perspective in ensuring yam improvememt in DR Congo is the dissemination of information or technology transfer. This is principally important for generating good and quality seedyam. In the presence of yam improvement program, efforts sould be made to organize trainings on technology transfer (yam minisett to begin with) on seedyam production for farmers’ through the extension experts as they are the closest to the farmers and most trusted by the farmers. Participatory plant breeding approach would also create an atmosphere for close relationship with the yam farmers as a means to rapidly understand their challenges.

## Conclusion

The study revealed a moderate diversity for yams across five different species in DR Congo. The diversity was relatively higher in Tshopo and Mongala provinces than Bas-Uélé province. The principal challenges limiting yam production in DR Congo surrounds the tuber quality attributes (poor post-harvest shelf life, tuber oxidation, poor taste, and rapid tuber hardiness) of the available varieties. Yam cultivation is targeted to meet the food and financial demands of the populace. Yam farmers preferred yams varieties with good tuber qualities, high yield and early maturing varieties. In the absence of formal seedyam production practice, farmers practiced the system of producing seeds by themselves as well as informal exchange of seeds with neighbors and friends. The establishment of a yam improvement program to meet farmers’ selection criteria, collection and conservation of yam germplasm, and the development of an effective seed delivery system to meet the seed availability and viability needs could increase yam production and profitability in DR Congo.

## Supplementary Information

Supplementary Tables.

## Data Availability

Data can be obtained upon request from the corresponding author.
