# Supplementary material for: Diversity, trait preferences, management and utilization of yams landraces (Dioscorea species): an orphan crop in DR Congo
Source: Sci Rep. 2022 Feb 10;12:2252. doi: 10.1038/s41598-022-06265-w (PMC8831613; doi:10.1038/s41598-022-06265-w)
Supplement: Supplementary file 1 — Supplementary Tables. [file 41598_2022_6265_MOESM1_ESM.docx]

**Diversity, trait preferences, management and utilization of yams landraces (*Dioscorea species*): an orphan crop in DR Congo**

**Idris I. Adejumobi^1,2^, Paterne A. Agre^2*^, Didy O. Onautshu^1^, Joseph G. Adheka^1^, Mokonzi G. Bambanota^1^, Jean-Claude L. Monzenga^3^, Joseph L. Komoy^1^, and Inacio M. Cipriano^1^**

^1^Department of Biotechnology, Faculty of Science, University of Kisangani, Kisangani, DR Congo; [adejumobiidris@yahoo.com](mailto:adejumobiidris@yahoo.com) (I.I.A); [didyonautshu@yaoo.com](mailto:didyonautshu@yaoo.com) (D.O.O); [jadheka@yahoo.fr](mailto:jadheka@yahoo.fr) (J.G.A) [gratien.mokonzi@unikis.ac.cd](mailto:gratien.mokonzi@unikis.ac.cd) (M.G.B); [cipriano12cipriano@gmail.com](mailto:cipriano12cipriano@gmail.com) (I.M.C); [josephkomoy@gmail.com](mailto:josephkomoy@gmail.com) (J.L.K)

^2^International Institute of Tropical Agriculture; [P.Agre@cgiar.org](mailto:P.Agre@cgiar.org) (P.A.A)

^3^Institut Facultaire des Sciences Agronomiques de Yangambi (IFA-YBI) à BP 1232 Kisangani DR Congo ; [claumonz@yahoo.fr](mailto:claumonz@yahoo.fr)

*Correspondence: [P.Agre@cgiar.org](mailto:P.Agre@cgiar.org)

**Supplementary tables**

**Sup Table 1. Ecological characteristics of the survey regions**

| **Province** | | **Territory** | **Coordinates (lat., long.)** | **Elevation (meters)** | **Mean Rainfall (mm/year)** | **Mean Temperature (°C)** | **Vegetation type** | | **Crops cultivation** |  |  |
| --- | --- | --- | --- | --- | --- | --- | --- | --- | --- | --- | --- |
| **Bas-Uélé** | | **Bambesa** | 3.44° N, 25.69° E | 415 | 1,716 | Min = 19 Max = 37 | Dense humid forest, Secondary forest, Forest galleries, Wooded savannah | | **Cereals** (Maize, Rice, Millet and Sorghum); **Root and Tubers** (Manioc, Yam, Potatoes and Taro); **Legumes** (Bean, Cowpea and Peas); **Oilseeds** (Peanut and Soya); **Fruits** (Banana and Plantain) |  |  |
|  |  | **Buta** | 2.81° N, 24.75° E | 430 | 2,374 | Min = 20 Max = 37 |  |  |  |  |  |
| **Mongala** | | **Bumba** | 2.20° N, 22.47° E | 409 | 2,424 | Min = 21 Max = 37 | Dense humid forest, Secondary forest, Edaphic Forest | | **Cereals** (Maize, Rice, Millet and Sorghum); **Root and Tubers** (Manioc, Yam, Potatoes and Taro); **Legumes** (Bean, Cowpea and Peas); **Oilseeds** (Peanut and Soya); **Fruits** (Banana and Plantain) |  |  |
|  |  | **Lisala** | 2.17° N, 21.49° E | 418 | 2,340 | Min = 21 Max = 37 |  |  |  |  |  |
| **Tshopo** | | **Kisangani** | 0.52° N, 25.20° E | 447 | 3,156 | Min = 21 Max = 39 | Dense humid forest, Secondary forest, Edaphic Forest | | **Cereals** (Maize, Rice, Millet and Sorghum); **Root and Tubers** (Manioc, Yam, Potatoes and Taro); **Legumes** (Bean, Cowpea and Peas); **Oilseeds** (Peanut and Soya); **Fruits** (Banana and Plantain) |  |  |
|  |  | **Isangi** | | 0.78° N, 24.27° E | 435 | 2,258 |  |  | Min = 21 Max = 35 |  | |

**Source:** National Agency for the Promotion of Investments (DR Congo); World Weather online (https://www.worldweatheronline.com)

**Sup Table 2: Landrace diversity across province, territory and village in the surveyed area**

| **Province** | **Territory** | **Village** | **Landrace richness** | **Shannon (H')** | **H_max** | **Equitability**  **(E)** |
| --- | --- | --- | --- | --- | --- | --- |
|  |  | Adiwaya | 2 | 0.30 | 0.69 | 0.44 |
|  |  | Bango | 3 | 0.89 | 1.10 | 0.81 |
|  |  | Bombele | 3 | 0.89 | 1.10 | 0.81 |
|  |  | Bongenge | 2 | 0.60 | 0.69 | 0.86 |
|  |  | Bulusu | 4 | 1.12 | 1.39 | 0.81 |
|  |  | Dingima | 2 | 0.45 | 0.69 | 0.65 |
|  |  | Mendigba | 2 | 0.66 | 0.69 | 0.95 |
|  |  | Mupembe | 4 | 1.09 | 1.39 | 0.79 |
|  |  | Ngbonga | 4 | 1.28 | 1.39 | 0.92 |
|  | **Bambesa** |  | **7** | **1.45** | **1.95** | **0.75** |
|  |  | Baebona | 5 | 1.48 | 1.61 | 0.92 |
|  |  | Bobanabendea | 5 | 1.31 | 1.61 | 0.82 |
|  |  | Bobomale | 2 | 0.64 | 0.69 | 0.92 |
|  |  | Bonzo | 2 | 0.54 | 0.69 | 0.78 |
|  |  | Boyelia | 2 | 0.64 | 0.69 | 0.92 |
|  |  | Kumu | 3 | 0.88 | 1.10 | 0.80 |
|  |  | Monjila | 3 | 0.85 | 1.10 | 0.77 |
|  |  | Q.Bale | 4 | 1.11 | 1.39 | 0.80 |
|  |  | Sombo | 3 | 0.82 | 1.10 | 0.75 |
|  | **Buta** |  | **7** | **1.39** | **1.95** | **0.71** |
| **Bas-Uélé** |  |  | **10** | **1.67** | **2.30** | **0.73** |
|  |  | Bongolo-II | 4 | 1.12 | 1.39 | 0.81 |
|  |  | Bonzo | 7 | 1.82 | 1.95 | 0.93 |
|  |  | Botsholi-I | 3 | 1.08 | 1.10 | 0.98 |
|  |  | Botsholi-II | 3 | 0.60 | 1.10 | 0.55 |
|  |  | Yamaluka-II | 3 | 0.92 | 1.10 | 0.83 |
|  |  | Yamoguo | 4 | 1.35 | 1.39 | 0.98 |
|  |  | Yamolea-I | 4 | 1.12 | 1.39 | 0.80 |
|  |  | Yanjumbu | 5 | 1.30 | 1.61 | 0.81 |
|  |  | Yapembe | 4 | 1.12 | 1.39 | 0.81 |
|  | **Bumba** |  | **15** | **2.42** | **2.71** | **0.89** |
|  |  | Bobi | 6 | 1.66 | 1.79 | 0.92 |
|  |  | Bokutu | 7 | 1.67 | 1.95 | 0.86 |
|  |  | Bosokuluki-I | 3 | 1.00 | 1.10 | 0.91 |
|  |  | Bosokuluki-II | 4 | 1.09 | 1.39 | 0.79 |
|  |  | Botukwa | 6 | 1.40 | 1.79 | 0.78 |
|  |  | Dika | 9 | 2.00 | 2.20 | 0.91 |
|  |  | Liweya | 6 | 1.52 | 1.79 | 0.85 |
|  |  | Mapasa | 5 | 1.47 | 1.61 | 0.92 |
|  |  | Ngunzibalele | 5 | 1.47 | 1.61 | 0.91 |
|  | **Lisala** |  | **16** | **2.22** | **2.77** | **0.80** |
| **Mongala** |  |  | **26** | **2.77** | **3.26** | **0.85** |
|  |  | Lilanda | 4 | 1.08 | 1.39 | 0.78 |
|  |  | Q. Bangala | 10 | 2.20 | 2.30 | 0.96 |
|  |  | Q. Lumumba | 10 | 1.91 | 2.30 | 0.83 |
|  |  | Yakako-I | 3 | 1.06 | 1.10 | 0.96 |
|  |  | Yakpondi | 4 | 1.27 | 1.39 | 0.91 |
|  |  | Yalibua | 4 | 1.35 | 1.39 | 0.97 |
|  |  | Yalinga | 8 | 1.80 | 2.08 | 0.87 |
|  |  | Yaondolo-II | 4 | 1.33 | 1.39 | 0.96 |
|  |  | Yaselia | 4 | 1.23 | 1.39 | 0.89 |
|  | **Isangi** |  | **20** | **2.32** | **3.00** | **0.78** |
|  |  | Babugana | 5 | 1.33 | 1.61 | 0.82 |
|  |  | Batikayafi | 4 | 1.07 | 1.39 | 0.77 |
|  |  | Likenga | 3 | 0.60 | 1.10 | 0.55 |
|  |  | Lugnunga | 6 | 1.58 | 1.79 | 0.88 |
|  |  | Magbololo | 4 | 0.98 | 1.39 | 0.71 |
|  |  | Maleke | 7 | 1.53 | 1.95 | 0.79 |
|  |  | Ngenengene | 4 | 1.21 | 1.39 | 0.87 |
|  |  | Ngenengene-II | 4 | 1.32 | 1.39 | 0.95 |
|  |  | Osio | 5 | 1.38 | 1.61 | 0.86 |
|  | **Kisangani** |  | **21** | **2.60** | **3.04** | **0.85** |
| **Tshopo** |  |  | **35** | **2.79** | **3.56** | **0.79** |
|  |  | **Average** |  | **1.32** | **1.58** | **0.84** |
